# The effects of prefrontal vs. parietal cortex transcranial direct current stimulation on craving, inhibition, and measures of self-esteem

**DOI:** 10.3389/fnins.2022.998875

**Published:** 2022-10-31

**Authors:** Milos Ljubisavljevic, Jonida Basha, Fatima Y. Ismail

**Affiliations:** ^1^Department of Physiology, College of Medicine and Health Sciences (CMHS), United Arab Emirates University, Al Ain, United Arab Emirates; ^2^Department of Pediatrics, College of Medicine and Health Sciences (CMHS), United Arab Emirates University, Al Ain, United Arab Emirates

**Keywords:** craving, transcranial direct-current stimulation (tDCS), dorsolateral prefrontal cortex (DLPFC), inhibition, self-esteem, body appreciation, inferior parietal lobule (IPL)

## Abstract

While prefrontal cortex dysfunction has been implicated in high food cravings, other cortical regions, like the parietal cortex, are potentially also involved in regulating craving. This study explored the effects of stimulating the inferior parietal lobule (IPL) and dorsolateral prefrontal cortex (DLPFC) on food craving state and trait. Transcranial direct current stimulation (tDCS) was administered at 1.5 mA for 5 consecutive days. Participants received 20 min of IPL, DLPFC, or sham stimulation (SHAM) each day which consisted of two rounds of 10-min stimulation, divided by a 10-min mindfulness task break. In addition, we studied inhibition and subjective psychological aspects like body image and self-esteem state and trait. To decompose immediate and cumulative effects, we measured the following on days 1 and 5: inhibition through the Go/No-go task; and food craving, self-esteem, and body appreciation through a battery of questionnaires. We found that false alarm errors decreased in the participants receiving active stimulation in the DLPFC (DLPFC-group). In contrast, false alarm errors increased in participants receiving active stimulation in the IPL (IPL-group). At the same time, no change was found in the participants receiving SHAM (SHAM-group). There was a trending reduction in craving trait in all groups. Momentary craving was decreased in the DLPFC-group and increased in IPL-group, yet a statistical difference was not reached. According to time and baseline, self-esteem and body perception improved in the IPL-group. Furthermore, self-esteem trait significantly improved over time in the DLPFC-group and IPL-group. These preliminary results indicate that tDCS modulates inhibition in frontoparietal areas with opposite effects, enhancing it in DLPFC and impairing it in IPL. Moreover, craving is moderately linked to inhibition, self-esteem, and body appreciation which seem not to be affected by neuromodulation but may rely instead on broader regions as more complex constructs. Finally, the fractionated protocol can effectively influence inhibition with milder effects on other constructs.

## Introduction

Eating is an essential human function linked to survival, pleasure, and reward. An important driving factor of eating is craving, a desire to consume a specific food that is difficult to ignore or satisfy by consuming an alternative (Martin et al., 2011). Food craving has been associated with eating disorder psychopathologies (Davis et al., 2011; Chao et al., 2016; Parker et al., 2021), weight gain (Boswell and Kober, 2016), and addiction relapse (Koob and Volkow, 2010). Therefore, it is essential to study food cravings in the context of preventing obesity and designing efficient interventions, especially when considering the effect of the recent lockdown on eating behaviors (Bhutani et al., 2021).

The research literature exploring brain centers regulating craving behavior focuses on the dorsolateral prefrontal cortex (DLPFC) as one of the critical nodes controlling eating behavior (Lefaucheur et al., 2017; Mostafavi et al., 2020; Saruco and Pleger, 2021). It has been suggested that the involvement of the DLPFC in eating behavior (Gomis-Vicent et al., 2019) is linked to its regulation of reward (McClure et al., 2004; Beaver et al., 2006), decision making (Knoch et al., 2006), and inhibitory control functions (Sridharan et al., 2008). In addition, the prefrontal cortex (PFC) typically orchestrates the brain resources to achieve a specific goal by guiding the activity of more posterior or subcortical areas (Miller and Cohen, 2001)and possibly automating these processes. This points toward the existence of a frontoparietal network, including areas like the DLPFC and inferior parietal lobule (stocktickerIPL) (Fincham et al., 2001; Dosenbach et al., 2007). It has been reported that parietal areas are involved in craving recreational drugs (Garavan et al., 2000; Noori et al., 2016), tobacco (Due et al., 2002; Ghahremani et al., 2018), alcohol (Schacht et al., 2013), and food (Giuliani and Pfeifer, 2015; Han et al., 2018; Stopyra et al., 2021). Furthermore, research combining functional magnetic resonance imaging (fMRI) and machine learning found that connectivity based models outperformed models limited to specific regional activity. This suggest that the regulation of craving is more strongly associated with interactions between brain regions compared to isolated regional activities (Kulkarni et al., 2022).

A recent meta-analysis indicated a more robust effect of right DLPFC stimulation on food craving reduction compared to the left side (Ester and Kullmann, 2022). This seems consistent with the right brain hypothesis of obesity (Alonso-Alonso and Pascual-Leone, 2007), which regards the right PFC as an important area for controlling food intake. Other theories have pointed out the imbalance between activity in the left and right frontal cortex, which is commonly referred to as asymmetric frontal cortical activity. According to this, right frontal cortical activity is associated with avoidance motivation, while left frontal cortical activity is associated with approach (Schutter et al., 2008; Kelley et al., 2017; McGeown and Davis, 2018). As claimed by this theory, stimulating the right side could result in a reduction of craving. Furthermore, attentional bias toward food has been linked with left frontal asymmetry (McGeown and Davis, 2018). While non-invasive brain stimulation (NIBS) has previously reduced the approach motivation and food craving by targeting the right prefrontal areas (Fregni et al., 2008). Regarding the parietal regions, electroencephalography (EEG) studies have linked resting-state right-sided alpha parieto-occipital activation to reduced hedonic food appreciation (van Bochove et al., 2016). Overall, evidence indicates that neuromodulation of the right DLPFC activity effectively reduces food cravings, and that the effects may outlast the intervention. Currently, research has not explored the effects of right IPL neuromodulation as part of a frontoparietal network in food craving.

Over the past decade, several novel approaches emerged, focusing on the modulation of DLPFC activity to enhance the control and reduction of craving (Gluck et al., 2017). Transcranial direct current stimulation (tDCS) is an extensively used neuromodulation technique that delivers a mild electrical current to the cortex via electrodes attached to the scalp (Vance et al., 2016). This electrical current passes through the skull, modifies neuronal trans-membrane potentials (Boes et al., 2018), and ultimately affects neuroplasticity (Nitsche et al., 2008). This method has shown varying degrees of therapeutic effects on multiple neurodegenerative diseases (Brunoni et al., 2016; Saxena and Pal, 2021). Furthermore, tDCS applied over the DLPFC can induce an immediate reduction of acute food craving (Goldman et al., 2011; Martin et al., 2011; Montenegro et al., 2012; Gluck et al., 2015) and elicit long-lasting effects which exceed the intervention (Ljubisavljevic et al., 2016).

One of the critical factors interacting and influencing craving is inhibition. According to the VandenBos and American Psychological Association (2015), inhibition implies the active blocking or delay of a response to a stimulus. The lack of inhibition has been associated with various addictions (Goldstein and Volkow, 2002; Volkow et al., 2016) and poor dietary habits (Mobbs et al., 2011; Forcano et al., 2018; Jones et al., 2018). Prefrontal areas are also established as a hub of inhibitory control (Goldstein and Volkow, 2011; Carr et al., 2021). Most NIBS studies investigating inhibition targeted areas such as the inferior frontal gyrus, DLPFC, and the orbital frontal cortex (Sarkis et al., 2014; Schluter et al., 2018; de Boer et al., 2021). Inhibition has also been linked to parietal brain regions (Menon et al., 2001; Osada et al., 2019; Kolodny et al., 2020; Ouerfelli-Ethier et al., 2021). This highlights the existence of a frontoparietal network that relates to executive control processes (Barbey et al., 2012; Niendam et al., 2012) and inhibition (Miyake et al., 2000).

Craving and obesity have been associated with a general sense of wellbeing (Vallis, 2019) and a physical appearance subdomain of self-esteem (Griffiths et al., 2010), possibly through cognitive biases related to the selective interpretation of body size and shape (Williamson et al., 1999). Empirical data have shown that body image bias is present in healthy subjects (Gagne et al., 2012; Cooper and Wade, 2015) and those with eating pathologies (van den Eynde et al., 2011; Lewer et al., 2017; Strand et al., 2021). Moreover, studies have suggested an interplay of factors like higher BMI, low self-esteem, and weight bias internalization to food addiction (Pape et al., 2021).

Apart from craving and inhibition, frontoparietal areas have been linked to self-awareness (Lavarco et al., 2022), body awareness (Takeuchi et al., 2019), normal bodily self-consciousness (Igelström and Graziano, 2017), self-appraisal (Knyazev et al., 2021), and more specifically to self-esteem (Agroskin et al., 2014). Resting-state fMRI research has revealed positive relations between the DLPFC and trait self-esteem (Pan et al., 2016). Transcranial direct current stimulation (tDCS) of the prefrontal cortex has been found to improve self-esteem through the reduction of rumination (De Raedt et al., 2017), although with varying results (Longe et al., 2010; Jiang et al., 2018). The IPL has been related to self-appraisal (Delahoy et al., 2022) and conscious awareness of the self (Davey et al., 2016). It is also part of the default network (Di and Biswal, 2014) associated with self-referential processing (Raichle, 2015). Thus, it seems plausible to assume that neuromodulation may, by altering the activity of the DLPFC and the IPL, impact self-esteem, body appreciation, and inhibition which may, if affected, interact with craving.

In summary, we explored whether tDCS of the IPL and the DLPFC may be effective in modulating craving. We also hypothesized that neuromodulation of these areas would affect inhibition and constructs like self-esteem and body perception, by potentially interacting with food cravings in high-food-craving participants.

## Materials and methods

### Participants

Initially, recruitment was done through a broadcast email to all staff and students of UAEU and a poster displayed outside the lab. A total of 174 subjects responded to our email and completed the first screening questionnaire, 67 were eligible, and 29 subjects participated (Mage = 26.24, *SD* = 10.44, ranging from 18 to 55 years), 12 females.

Mean BMI was 25.72 kg/m^2^ (*SD* = 5.81, minimum value = 17.20, maximum value = 41.3). All the participants were naïve to tDCS stimulation. Power analysis was calculated through G-power 3.1.5 (Erdfelder et al., 2009), which estimated a total sample of 31 would be needed to reach a statistical power of 0.09, and significance was set at 0.05. For practical reasons related to the Covid pandemic, we reached a sample of 29 individuals. Exclusion criteria included: a history of neuropsychiatric or eating disorder, chronic disease, depression, seizures, migraines, pregnancy, alcohol or drug consumption, as well as contraindications for tDCS such as metal implants in the head, scars, surgeries, and previous loss of consciousness, while to be included subjects had to be right-handed and have a high level of craving as measured by reduced Food Cravings Questionnaire-Trait (FCQ-T) (Cepeda-Benito et al., 2000; Meule et al., 2014) as well as a craving visual analog scale (CVAS). Overall scores of both questionnaires ranged from 27 to 150, and the cutoff for inclusion in the study was a score of 100 and above.

All participants signed informed consent, and the study was approved by the HERTC (Human Ethics at UAEU–ERH-2019-5910).

### Measures

#### Pre-screening

All the initial subjects interested in this study were redirected to a screening questionnaire to collect categorical data such as age, height, weight, and gender. Thereafter the questionnaire continued, and they completed the reduced FCQ-T (Cepeda-Benito et al., 2000; Meule et al., 2014) and the CVAS. In the reduced version of FCQ-T, subjects had to answer 15 questions rated on a 6-point scale ranging from never = 1 to always = 6. Measurement of craving by CVAS is easy to collect and is easily quantified (McMillan and Gilmore-Thomas, 1996). During the CVAS questionnaire, participants were exposed to 12 food pictures (three sweets, three carbohydrates, three fast-food items, and three high fats). Participants had to rate “How much would you like to eat this” on a scale from 1 to 5. The pictures were taken from the foodcast research image database (Foroni et al., 2013), consisting of standardized pictures of food and non-food items.

#### Transcranial direct current stimulation protocol

We applied tDCS over 5 consecutive days, which was administered through a transcranial DC Stimulator (Model 1300A, Soterix Medical Inc., USA) connected to saline-soaked sponge pads (5 × 7 cm). The intensity was set to 1.5 mA with a ramp up and down of 30 s each and a current density of 0.04 mA/cm^2^.

Stimulation was divided into two sessions of 10 min each with a break of 10 min in between, creating a fractionated tDCS protocol. In the sham stimulation group (SHAM-group), after the initial ramp-up of 30 s, the current intensity was automatically set to zero for 10 min, ending with a ramp-down of 30 s.

The anode was placed over F4, according to the international 10–20 system, on participants who received DLPFC (DLPFC-group) and SHAM (SHAM-group) stimulation. DLPFC position near F4 has been supported by neuronavigation studies (Fitzgerald et al., 2009) and is constantly used in food craving studies (Sedgmond et al., 2019; Ghobadi-Azbari et al., 2022). While the anode was placed over P4 on participants who received IPL (IPL-group) stimulation (Herwig et al., 2003). This position is also widely used in non-navigated NIBS studies (Coulborn et al., 2020; Golaszewski et al., 2021). In all the groups, the cathode was placed in the left supraorbital area, FP1. The use of the 10–20 based system has a considerable degree of face validity as the system accounts for individual variation in head size that is not accounted or in other methods like 5 cm approach (Fitzgerald et al., 2009).

#### Go/No-go task

The task employed in study inhibition was the Go/No-go task (Bari and Robbins, 2013) which consisted of showing the participant 4 quadrants on a black screen. The letters “P” or “R” randomly appeared in a quadrant at a time. Subjects were instructed to press a button when they saw a “P” on the screen and refrain from pressing it when seeing the “R.” The order was reversed in the second half of the task. Before each task, there was a short training session. Inhibition parameters followed those described in Bezdjian et al. (2009). The Go/No-go task was administered on a computer through a free program, PEBL (Mueller and Piper, 2014). We analyzed the number of False Alarms (FA) as an indicator of inhibition by quantifying the number of errors when the button was pressed when it was not required.

#### Food cravings questionnaire-state and food cravings questionnaire-trait

The Food Cravings Questionnaire-State (FCQ-S) and FCQ-T are two psychometrically sound measures to quantify an individual’s level of food craving, with excellent internal consistency and construct validity (Cepeda-Benito et al., 2000).

The FCQ-T questionnaire consists of 39 statements measuring the stability of craving features over time and situations. Subjects had to rate each statement on a 6-point scale ranging from never = 1 to always = 6, where higher scores indicate a higher level of craving. It measures nine dimensions such as intention and planning to eat, the anticipation of positive reinforcement from eating, anticipation of relief from negative feelings, lack of control, overeating, preoccupation with food, craving as a physiological state, and emotions before or during craving or eating, environmental triggers of craving and guilt resulting from giving in to cravings. Overall, Cronbach’s alpha is 0.97 (Cepeda-Benito et al., 2000).

The FCQ-S questionnaire measures the contextual states of craving. It has five subscales: the desire to eat, the anticipation of positive reinforcement from eating, relief from negative states resulting from eating, obsession over food or lack of control over overeating, and craving as a physiological state. Subjects had to evaluate each statement on a 5-point scale ranging from strongly disagree = 1 to strongly agree = 5. The reported Cronbach’s alpha is 0.94 (Cepeda-Benito et al., 2000).

#### Body appreciation scale-2

We selected The Body Appreciation Scale-2 (BAS-2) questionnaire (Tylka and Wood-Barcalow, 2015), a revised version of the Body Appreciation Scale (BAS) (Avalos et al., 2005), to measure personal appreciation and positive attitude toward one’s body. It consists of 10 statements, which subjects had to rate on a scale ranging from 1 = never to 5 = always, measuring dimensions like body esteem, body surveillance, body shame, and psychological wellbeing. Higher scores indicate higher body appreciation. A review of body image measures reported strong internal consistency for BAS-2, with nearly all studies reporting Cronbach’s alpha ≥0.70 (Kling et al., 2019). It significantly correlated with other body image and well-being measures like self-esteem (Kling et al., 2019).

#### Revised Janis and Field feelings of inadequacy

We used a revised version of the Janis & Field Feelings of Inadequacy (Janis and Field, 1959; Fleming and Courtney, 1984). It comprises 36 items, measuring general self-esteem where participants reply choosing from a scale ranging from very often/confident = 1 to not at all often/confident = 5. Higher scores reveal higher self-esteem. Measured factors include self-regard, social confidence, school abilities, physical appearance, and physical abilities. The reliability estimate is 0.91 (Fleming and Courtney, 1984). It is suitable for examining multiple components of self-esteem (Heatherton and Wyland, 2003). For ease, we will refer to this as SE-T (self-esteem trait).

#### The state self-esteem scale

The State Self-Esteem Scale (SSES) measures momentary self-esteem fluctuations. The questionnaire comprises 20 statements scored on a 5-point scale (not at all = 1 to extremely = 5), measuring three dimensions: performance, social, and appearance (Hetherton and Polivy, 1991). Higher scores imply higher self-esteem. We chose this questionnaire, expecting it would be more sensitive to immediate changes resulting from the stimulation, complying with the state FCQ (Cepeda-Benito et al., 2000). The coefficient alpha is 0.92 (Hetherton and Polivy, 1991). For ease, we will refer to this as SE-s (self-esteem state).

### COVID precautions

The questionnaires mentioned above and the cognitive tasks were administered online to reduce the amount of physical paper used. In addition, we observed physical distancing, used personal protective equipment such as gloves and masks, and controlled entrance to the campus premises upon proof of a negative PCR report and body temperature check. Furthermore, research equipment was disinfection before and after use by following the guidelines for good practice and safety during the COVID-19 pandemic (Bikson et al., 2020).

### Study procedure

After completing the initial inclusion questionnaire online, accepted participants were invited to the lab for 5 consecutive days, 2–3 hours after their last meal. On the first day, they signed the informed consent, were screened for inclusion criteria, filled the batteries of questionnaires, and completed the Go/No-go task. They then received tDCS stimulation, and repeated the questionnaires and the Go/No-go task. They also indicated stimulation after-effects on a scale from 1 to 10 for dimensions like headache, neck pain, back pain, blurred vision, scalp irritation, tingling, itching, burning sensation, dizziness, acute mood change, increased heart rate, and anxiety. Finally, they had to reveal whether they believed they were receiving the active or sham stimulation. The protocol was repeated on the last day, day 5. On the other 3 days, tDCS was administered, and the adverse effects and blinding effectiveness were measured. Stimulation was administered for 10-min, followed by a 10-min break, and concluded with another 10-min stimulation. Participants were given a mindfulness script which they had to read and think about during the 10-min break. This was done to obtain standardization over all the groups. Upon completion of the study, participants were debriefed and received a token of appreciation.

### Statistical method

Analysis was performed using IBM^®^ SPSS^®^ software. For the main analysis, we chose Generalized Estimating Equation (GEE). This method is adequate for non-normally distributed data and is robust against choosing the wrong correlation structure (Hubbard et al., 2010). The predictors were stimulation, modeled as a factor, and time and baseline as the covariates for each measured outcome (FCQ-T, FCQ-S, SSES, SE-T, BAS-2, FA). The SHAM-group data were used as a reference in all analyses. We computed the main effects and two-way and three-way interaction between all the predictors to better understand if outcomes would change depending on baseline values. As data were not normally distributed, we chose Gamma with Log link. Alpha level was set at 0.05.

## Results

### Demographic data

Where the Shapiro-Wilks test revealed non-normally distributed data, we adopted the Kruskal-Wallis H test or Fisher’s exact test. Otherwise, we used one-way ANOVA to compare groups. There was no difference in terms of age [*X*^2^(2) = 3.249, *p* = 0.197], BMI [*F*(2, 26) = 0.230, *p* = 0.796], gender (Fisher’s exact test, *p* = 0.452), or baseline as shown in Table 1. The only exception was the inhibition FA, where baseline values were significantly different between groups [*F*(2, 26) = 6.056, *p* = 0.007]. For this reason, we choose to enter baseline as a moderator in all the analyses.

**TABLE 1:** Demographic data.

	DLPFC (*n* = 9)	IPL (*n* = 10)	SHAM (*n* = 10)	*p*-value
Age	29.4 ± 11.9	24 ± 9.8	25.6 ± 9.9	0.197
BMI	26.1 ± 8.2	24.6 ± 5.5	26.3 ± 3.5	0.796
Gender (F/M)	2/7	5/5	5/5	0.452
Baseline FCQT	176.8 ± 27.4	166.4 ± 28.7	176.8 ± 34.1	0.682
Baseline FCQS	57.2 ± 10.2	50.7 ± 15.8	53.3 ± 12.2	0.562
Baseline SE-T	112.1 ± 23.6	121.8 ± 22.5	113.4 ± 20.9	0.589
Baseline SE-S	71.7 ± 13.3	72.8 ± 13.5	66.7 ± 10.3	0.515
Baseline BA	37.5 ± 8.3	33.4 ± 7.8	36.3 ± 7.6	0.511
Baseline FA	14.2 ± 5.6	6.4 ± 4	8.3 ± 5.4	0.007

### Blinding

It was a single-blind study where the experimenter was aware of the stimulation group, but the participants were not. Most participants guessed that they were in the active group, and their guesses did not differ between groups *x*^2^(2) = 4.988, *p* = 0.083. In the SHAM-group, 68% guessed wrong. Thus, blinding was effective for this group. Overall, 65% guessed right, while 34% guessed wrong over all the groups.

### Side effects

For each side effect, we calculated a mean score of the ratings over the 5 days. Subsequently, we conducted the Independent-Samples Kruskal-Wallis Test since data were not normally distributed. Groups did not differ in adverse effects, which were mild (not greater than 3.8 out of 10).

### Main analysis

#### Food cravings questionnaire-trait and food cravings questionnaire-state

For trait craving, raw data showed a decrease in all groups across days (Table 2). However, the effect was insignificant (Wald *X*^2^ = 5.235, *p* = 0.073), as shown in Table 3.

**TABLE 2:** Craving, self-esteem, body appreciation questionnaires scores as well as results of Go/No-go task.

		Day 1 pre	Day 1 post	Day 5 pre	Day 5 post
FCQT	DLPFC	176.8 ± 27.4	164.8 ± 33	154.8 ± 28.1	154.8 ± 24.1
	IPL	166.4 ± 28.7	159.6 ± 24.6	152 ± 39.4	147.5 ± 34.8
	SHAM	176.8 ± 34.1	167.3 ± 25.7	163.5 ± 34.3	158.1 ± 39.2
FCQS	DLPFC	57.2 ± 10.2	56.7 ± 13.6	50.5 ± 13	48.6 ± 15.4
	IPL	50.7 ± 15.8	54.5 ± 7.9	53.3 ± 10.7	53.9 ± 8.8
	SHAM	53.3 ± 12.2	57.3 ± 8.5	57.7 ± 10.5	50.1 ± 14
SE-T	DLPFC	112.1 ± 23.6	115.5 ± 25.7	118.7 ± 21.4	118.2 ± 21.8
	IPL	121.8 ± 22.5	123.8 ± 20.7	126.6 ± 23.6	131.2 ± 20.6
	SHAM	113.4 ± 20.9	113.1 ± 14.2	123.3 ± 17.7	127.7 ± 22.8
SE-S	DLPFC	71.7 ± 13.5	75.3 ± 12.6	71.8 ± 12.8	71.5 ± 12.9
	IPL	72.8 ± 13.5	71.9 ± 14.7	76 ± 14.5	75.1 ± 13.4
	SHAM	66.7 ± 10.3	69.9 ± 8.9	71.8 ± 11.8	72.9 ± 11.6
BA	DLPFC	37.5 ± 8.3	36.5 ± 9.7	34.6 ± 8.5	35.7 ± 7.5
	IPL	33.4 ± 7.8	34.4 ± 10.1	36.1 ± 11	36.2 ± 11.3
	SHAM	36.3 ± 7.6	37.1 ± 6.9	38.5 ± 6.5	40 ± 6.8
FA	DLPFC	14.2 ± 5.6	13.3 ± 6.4	8.8 ± 4.7	9.6 ± 2.6
	IPL	6.4 ± 4	7.8 ± 5.4	9.7 ± 7.1	12.6 ± 8.5
	SHAM	8.3 ± 5.4	9.7 ± 7.1	9.8 ± 8.1	10.6 ± 7.8

**TABLE 3:** Results of the general estimating equations.

	FCQT		FCQS		SET	
			
	Estimate	*p*	Estimate	*p*	Estimate	*p*
Time	−0.024	<0.001	0.146	0.394	−0.079	0.133
DLPFC	−0.017	0.843	−1.016	0.164	−0.727	0.004*
IPL	0.010	0.851	0.611	0.080	−0.711	0.024*
Baseline	0.005	<0.001*	0.009	0.190	0.003	0.026*
DLPFC × time	0.278	0.022	0.480	0.262	0.228	0.013*
IPL × time	0.046	0.799	−0.252	0.158	0.479	0.032*
DLPFC × baseline	−0.003	0.034	0.017	0.240	0.007	0.002*
IPL × baseline	0.000	0.888	−0.014	0.052	0.006	0.009*

	**SES**		**BA**			**FA**
			
	**Estimate**	***p***	**Estimate**	***p***	**Estimate**	***p***

Time	0.073	0.300	0.208	0.006*	0.350	0.009*
DLPFC	0.015	0.905	−0.431	0.085	0.917	0.017*
IPL	−0.685	<0.001*	−0.712	0.007*	−0.609	0.105
Baseline	0.012	<0.001*	0.026	0.000*	0.153	<0.001*
DLPFC × time	−0.040	0.666	−0.027	0.805	−0.275	0.155
IPL × time	0.289	0.023*	0.179	0.444	0.321	0.153
DLPFC × baseline	0.000	0.790	0.011	0.070	−0.082	0.037*
IPL × baseline	0.009	<0.001*	0.021	0.005*	0.068	0.111

Only for marked values, there was a general effect in the test of model effects.

For the craving state, there was a decreasing trend in craving state scores in the DLPFC-group, while in the IPL-group, there was an increase. In the SHAM-group, the scores initially increased and then decreased. GEE revealed an effect of stimulation (Wald *X*^2^ = 8.301, *p* = 0.016), baseline (Wald *X*^2^ = 4.063, *p* = 0.044) as well as an interaction between stimulation and baseline (Wald *X*^2^ = 8.939, *p* = 0.011). *Post-hoc* tests did not detect any significance.

#### Self-esteem trait and state self-esteem scale

Regarding self-esteem trait, the raw data showed a general increase in time across groups. GEE analysis revealed that main effect of baseline (Wald *X*^2^ = 4.967, *p* = 0.026) and effect of both DLPFC (Wald *X*^2^ = 8.319, *p* = 0.004) and IPL (Wald *X*^2^ = 5.100, *p* = 0.024), with a decrease in scores of self-esteem compared to the SHAM. However when we looked at interactions of DLPFC with time (Wald *X*^2^ = 63,213, *p* = 0.013), and IPL with time (Wald *X*^2^ = 4.594, *p* = 0.032) as well as the interaction of DLPFC with baseline (Wald *X*^2^ = 9.892, *p* = 0.002) and IPL with baseline (Wald *X*^2^ = 6.827, *p* = 0.009) there was a significant increase in self-esteem trait scores signifying improvement compared to SHAM.

Regarding self-esteem state, descriptive data showed a trend for increasing scores over time in IPL and SHAM but almost no change in DLPFC. In GEE, we found the main decrease in scores of IPL compared to SHAM (Wald *X*^2^ = 21.352, *p* < 0.001), but if we also take into consideration the two-way interaction with time in IPL (Wald *X*^2^ = 5.195, *p* < 0.023) or with baseline, scores of self-esteem in IPL were higher than SHAM (Wald *X*^2^ = 14.789, *p* < 0.001).

#### Body appreciation (body appreciation scale-2)

Exploratory data regarding body appreciation showed a decrease in DLPFC and an increase in IPL and SHAM. GEE revealed main effect of time (Wald *X*^2^ = 7.567, *p* = 0.006), main effect of baseline (Wald *X*^2^ = 73.250, *p* = 0.000) and decrease of BA scores in IPL (Wald *X*^2^ = 7.227, *p* = 0.007) compared to SHAM. However, in the interaction between IPL and baseline (Wald *X*^2^ = 7.833, *p* = 0.005), there was an improvement in BA compared to SHAM.

#### False alarms

**FIGURE 1:**
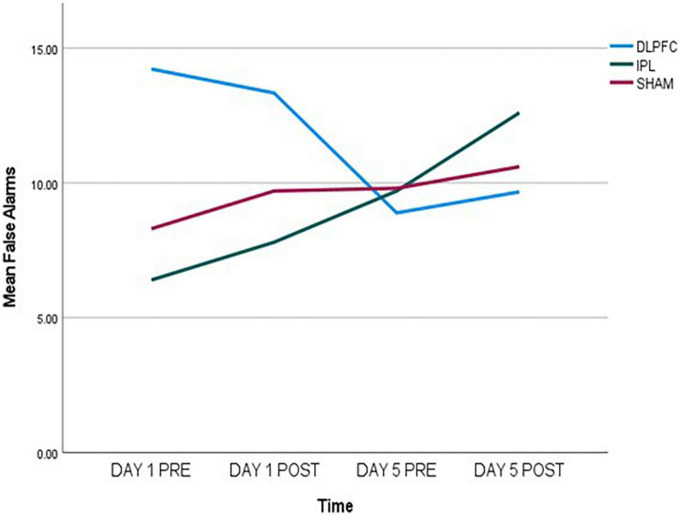
False alarms (FA) data, measured pre- and post-stimulation on day 1 and day 5, indicating a decrease in the DLPFC-group, an increase in the IPL-group, and almost no change in the SHAM-group.

Regarding false alarms (FA), exploratory data showed a decrease in errors in DLPFC, an increase in IPL, and almost no change in SHAM as shown in Figure 1. In GEE analysis we found increase of errors across time (Wald *X*^2^ = 6.790, *p* = 0.009), and effect of baseline (Wald *X*^2^ = 22.695, *p* < 0.001), and more errors in DLPFC compared to SHAM (Wald *X*^2^ = 5.663, *p* = 0.017). However, when we looked at two-way interactions between DLPFC and baseline (Wald *X*^2^ = 4.343, *p* = 0.037), there was a decrease in false alarms signifying improvement compared to SHAM.

Effects of Go/No-go task were stronger compared to other outcomes. Paired-samples *post-hoc* t-test showed that in either DLPFC and IPL there was significant change from day 1 pre-stimulation to day 5 after stimulation [respectively, *t*(8) = 2.705, *p* = 0.027 and *t*(9) = −2.680, *p* = 0.025], while there was no significant change in SHAM [*t*(9) = −1.017, *p* = 0.336].

## Discussion

The present study sought to investigate whether the geography of cortical stimulation sites could be extended beyond the DLPFC to the IPL area, thus underpinning its involvement in orchestrating control of food cravings. Furthermore, to investigate whether the stimulation of these sites alters control of inhibition, self-esteem, body appreciation, and their interactions with cravings.

### Main aim—Transcranial direct current stimulation effect on food craving and expanding of stimulation sites

Concerning the first aim, the effects of tDCS trended regarding the craving trait. These results did not replicate the general effects of DLPFC stimulation on craving (Fregni et al., 2008; Lefaucheur et al., 2017; Ghobadi-Azbari et al., 2022). However, inconclusive results have been reported in literature, possibly due to variation in stimulation paradigms [see Ester and Kullmann, 2021 for a meta-analysis].

We also found a trend for craving state to be reduced in DLPFC-group and increased in the IPL-group. We speculate that this effect can be linked to the negative relation between IPL stimulation and inhibition as opposed to the enhancement of inhibition in the DLPFC-group. In the case of food cravings, the results might not have reached significance due to the low stimulation intensity and the application of a spaced-stimulation protocol. Furthermore, due to the practical inclusion criteria and challenges related to the COVID pandemic, our sample group comprised of moderate craving subjects. Studies have shown that eating behavior traits significantly predict tDCS effectiveness (Beaumont et al., 2022).

Contradicting our hypothesis, the frontal and parietal areas yielded contrary results. We stimulated the right hemispheres based on the assumption that left frontal activity is associated with approach motivation, while right frontal cortical activity is associated with avoidance motivation (Kelley et al., 2017; McGeown and Davis, 2018). Thus we hypothesized that stimulating the right hemisphere could potentially reduce craving. However, several studies have shown opposite activation of the anterior and posterior cortical regions (Morillas-Romero et al., 2013; van Bochove et al., 2016). van Bochove et al. (2016) reported that resting-state EEG was only linked to the hedonic valuation of food in the parieto-occipital sites and not in the anterior areas. This suggests a functional difference in the lateralization, possibly due to the top-down specialization of the frontal areas as opposed to the more bottom-up properties of the parietal regions (Li et al., 2010).

Finally, there might also be a differential effect of cue-induced as opposed to an abstinence-induced craving, i.e., craving elicited spontaneously from refraining from substance consumption. It has been shown that abstinence-induced craving for smoking was correlated with resting-state cerebral blood flow to the right DLPFC and other areas but not to the parietal regions (Wang et al., 2007). This could possibly be due to abnormal thalamus-DLPFC connectivity linked to attentional biases and reward (Wang et al., 2018). In these studies, abstinence was defined by a 12-h gap from smoking, while our protocol included only 2–3 h of abstinence. Nevertheless, it can be assumed that there might be a difference as opposed to a cue-induced craving, even in our case.

### Secondary aim—Craving related to self-esteem, body appreciation, and inhibition

Despite the lack of a significant effect on craving, in this study, stimulation of the DLPFC and the IPL yielded effects on other constructs. This seems to imply that craving can be moderately linked to self-esteem, body appreciation, and inhibition since changes in these outcomes did not necessarily reflect changes in craving or changes of similar strength.

However, we noticed a general trend of an inverse relation between IPL stimulation and DLPFC stimulation, i.e., while DLPFC would inhibit, IPL would activate.

#### Self-esteem, body appreciation, and craving

Both stocktickerIPL and DLPFC areas were linked to self-esteem trait. This confirms a right hemispheric dominance observed in neural correlates of self-conscious emotions (Devinsky, 2000; Lavarco et al., 2022). Our findings agree with previous research on self-esteem testing through tDCS (Pan et al., 2016; De Raedt et al., 2017). As well as with tDCS studies linking specific prefrontal areas to factors like unfairness concerning the self (Civai et al., 2014), down-regulation of negative emotions (Peña-Gómez et al., 2011) and rumination (Allaert et al., 2021).

Stimulation of the stocktickerIPL did have a beneficial effect on self-esteem state and body appreciation. Studies show that the stocktickerIPL (Uddin et al., 2005) and the Temporoparietal Junction (TPJ) are related to a sense of self, self-consciousness, self-appraisal, and self-esteem (Knyazev et al., 2021). The authors suggest that the right stocktickerIPL might play a role in self-other discrimination through the frontoparietal mirror neuron network (Uddin et al., 2005). Transcranial direct current stimulation (tDCS) over the TPJ has also been related to mental representations of self and other people (for a review, see Sellaro et al., 2016 and references therein). Meanwhile, stimulation of the DLPFC did not affect state self-esteem, a finding more in line with studies showing a negative effect (Jiang et al., 2018). This might explain the contradicting effects found in the literature about the DLPFC and self-esteem, associated with different aspects of self-esteem, namely state or trait.

The self-esteem state seemed not dependent on craving. Changes after tDCS had the same direction and were increased in the IPL-group conditions and decreased or unaltered in the DLPFC-group. Regarding self-esteem and craving traits, there seemed to be a spontaneous improvement in both constructs in all groups, which was significant for self-esteem but insignificant in the case of craving. Body appreciation followed the same pattern as the self-esteem state, i.e., an increase in the IPL-group and a decrease in the DLPFC-group. In summary, this seems to support that self-esteem and body appreciation is independent of craving and reliant on a paradox of other factors. A more robust connection seems to exist in pathological eating behaviors (Pape et al., 2021).

#### Inhibition and craving

The DLPFC was more involved in inhibition. Stimulation significantly reduced FA, thus improving inhibition performance. This agrees with previous studies regarding the DLPFC and the PFC as the central hub of cognitive control and inhibition (Cieslik et al., 2013; Salehinejad et al., 2017; Maier et al., 2018; Lavarco et al., 2022). Though more pronounced in the DLPFC, there was also a correlation of the IPL with inhibition. However, it was the opposite effect leading to deterioration in performance. Previous studies have linked the frontoparietal network to inhibition (Garavan et al., 1999; Rubia et al., 2001, 2003; Niendam et al., 2012). Functional connectivity studies show that the frontal-parietal regions are involved in a central executive network activity during cognitive tasks (Seeley et al., 2007) and carry out top-down functions. However, in our study, the effect had the opposite direction. Research reported an effect of tDCS stimulation of the parietal cortex on the increase in the number of false recognitions related to memory (Pergolizzi and Chua, 2015). In this study, the authors attributed the effect to a possible reaction of parietal areas to the perceived oldness of a stimulus, irrespective of the accuracy in relation to the task. Other studies show that anodal tDCS stimulation of the right posterior parietal cortex disrupted the processing of a single stimulus in the contralateral field (Filmer et al., 2015), thus linking it with visuospatial attention. Finally, a study showed that during a task with targets and distractors, attention to highly visible non-targets deactivated parietal regions (Farooqui and Manly, 2018). This implies that in our Go/No-go task, the successful completion would rely on deactivating these areas during No-go cues. Since we artificially activated the IPL region through tDCS, we believe this led to more FA and, thus, more errors.

Concerning the craving state, there seemed to be a link to inhibition. The cravings decreased when inhibition improved, as in the DLPFC-group. Meanwhile, in the IPL-group, craving scores increased with an associated deterioration of inhibitory performance. This connection was not found for the craving trait. Since the craving state is a construct that captures the fluctuations of craving, it is more sensitive to stimulation, unlike the craving trait, which is supposed to be more rigid.

However, the lack of a strong connection between craving and inhibition is puzzling. Even though inhibition was significantly improved in the DLPFC-group and impaired in the IPL-group, this did not affect craving. This might be due to the difference between responders and non-responders potentially included in the study. We hypothesize that craving is a more subjective construct, operationalized through self-report, but might arguably be a more rigid construct. Which possibly relies on a more distributed network involving cortical and subcortical components, which could not be influenced by tDCS to the same extent as inhibition. Perhaps, a potentially stronger effect could have been found if the study focused on food-related inhibition and not on a general inhibition concept.

### Ancillary observations—spaced stimulation protocol

In this study, we applied a spaced stimulation protocol. The initial research which explored the effects of tDCS on craving applied a continuous stimulation protocol, which, although very well tolerated, was occasionally associated with discomfort or minor adverse effects (Matsumoto and Ugawa, 2017). The space stimulation protocol was based on previously reported observations (Monte-Silva et al., 2010, 2013), which indicated that applying a second stimulation while the after-effects of the first were still ongoing enhances the magnitude and duration of the tDCS-induced effects, and more so than merely prolonging the stimulation. Our results showed that a spaced protocol was effective in the case of inhibition. Monte-Silva et al. (2010, 2013) reported that while exploring a spaced protocol, they kept the stimulation intensity at 2 mA. We reduced the intensity to 1.5 mA to test an even milder protocol. Subsequently, our results showed stronger effects in inhibition measured through the Go/No-go task and milder effects in the other constructs, such as self-esteem and body appreciation. This might be because inhibition was measured more objectively through an operational task, while the other constructs were measured through self-report, thus being more prone to the effect of tiredness and boredom, not proper focus, and potential challenges related to language (del Boca and Noll, 2000; Stanton et al., 2002).

Our results provide a promising new avenue that is much easier for sensitive categories such as the elderly or those with a lower tolerance to potentially adverse effects.

### Limitations

There are potential limitations in this study. The study was conducted as a single-blinded, randomized, parallel-group trial, implying that the researcher administering the tDCS was aware of the modality (i.e., active vs. SHAM). This could potentially impact the results. However, a large study combining 146 meta-analyses has shown that biases associated with lack of blinding are more significant in trials with subjectively assessed outcomes as opposed to objective ones (Wood et al., 2008). The majority of the measures in our study were computer-based without any interference from the researchers. Thus, it is highly unlikely that the researcher’s expectations influenced the outcome (Wallace et al., 2016; Yavari et al., 2018).

Furthermore, the data for each participant were coded for the analysis, removing potential biases. It should also be noted that adequate blinding in tDCS experiments is also affected by other factors related to stimulation that inevitably give the experimenters access to the study group. These are related to skin redness because of stimulation, which has been shown to compromise the adequate blinding of participants (Woods et al., 2016) and the need for continuous monitoring of impedances to ensure effective stimulation. Finally, considering safety measures during the ongoing pandemic, we felt it safer to limit the participant’s contact with multiple researchers.

Another limitation may be related to the fact that despite our attempts to recruit a more inclusive cohort, our sample was comprised mainly of students and their relatives. We cannot discern to what extent it is representative of the population. We also did not explore the long-term effects (preceding 1 month) of tDCS or control factors like boredom.

It should also be noted that the absence of significant tDCS effects on craving may be related to other sources of variability often neglected in the literature (Vergallito et al., 2018) like stable and variable inter-individual differences, such as morphological, genetic, and hormonal features which may partially account for the heterogeneity of responses (i.e., responders vs. non-responders).

### Future directions

To our knowledge, this is the first study where a spaced protocol has been used. It was still effective despite being less “invasive.” Higher doses, higher current densities, and higher stimulation periods have been associated with effects of larger magnitude and duration (Brunoni et al., 2012), which can be associated with skin problems in some cases (Matsumoto and Ugawa, 2017). Decreasing the current might maintain or reduce theoretical risks (Bikson et al., 2016). It would be interesting to apply the same protocol to other operational tasks with established effects in the literature. To the best of our knowledge, this is the first time that outcomes such as self-esteem and body appreciation are studied in relation to the craving, examining the tDCS effects and aiding a better understanding of the complex construct such as craving.

Furthermore, it would be interesting to replicate the study with stronger parameters of tDCS like a 2 mA intensity or rTMS to explore these subjective dimensions. Alternatively, future studies could try and operationalize the same constructs differently rather than through questionnaires. Also, a direct comparison between this kind of stimulation and a continuous one might be informative. Finally, we believe that the results of this study prompt further exploration of the involvement of the DLPFC and the IPL in inhibition and to better understand the dynamics of the frontoparietal networks.

## Data availability statement

The original contributions presented in the study are included in the article/supplementary material, further inquiries can be directed to the corresponding author.

## Ethics statement

The studies involving human participants were reviewed and approved by the HERTC (Human Ethics at UAEU–ERH-2019-5910). The patients/participants provided their written informed consent to participate in this study.

## Author contributions

ML contributed to the conception and design of the study, and wrote the manuscript. JB collected the data and performed the statistical analysis. FI supervised the findings of this work. All authors contributed to manuscript revision, read and approved the submitted version.
